# Supplementary material for: Mutual generation in neuronal activity across the brain via deep neural approach, and its network interpretation
Source: Commun Biol. 2023 Oct 31;6:1105. doi: 10.1038/s42003-023-05453-2 (PMC10618281; doi:10.1038/s42003-023-05453-2)

Supplemental material

In this study, the data were divided into 16 groups according to the group classifications shown in Table 1. Two slices are included for each group.

Starting from the next page, the results of the 16 groups are summarized in 16 pages labeled as a-p. Each page includes three panels (a)-(c), respectively. (a) shows the angle of the slices cut out, and the MRI cross section is indicated by a black line. If the line appears to be a single line, it is the case that the angles of the slices coincide incidentally. In (b) and (c), the left and right images respectively show the distribution of neurons on the electrode (left) and the position of the slice on the atlas (right). The different colors of the markers in the left figure refer to the different cortical layers, and the Allen institute atlas is depicted in the picture of the MRI cross section on the right. The color of the marker in (b) distinguishes between subcortical or intracortical layers, but layer differences are not used in this study.

a. Left Occipital

(a)

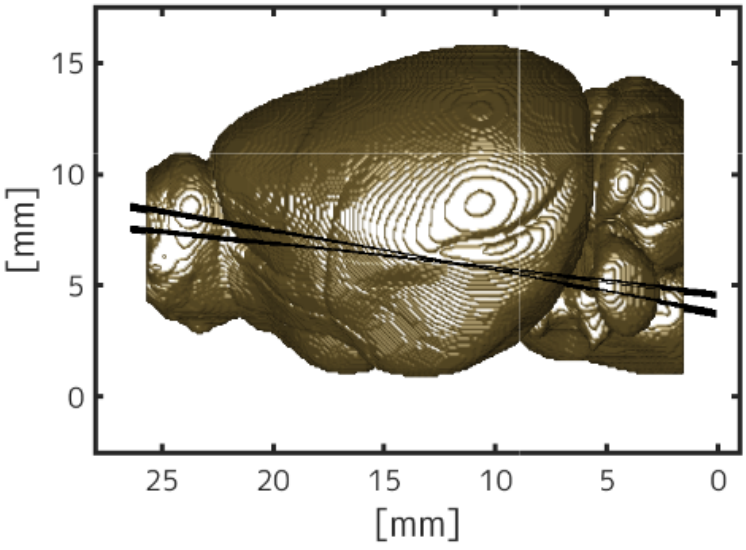

(b)

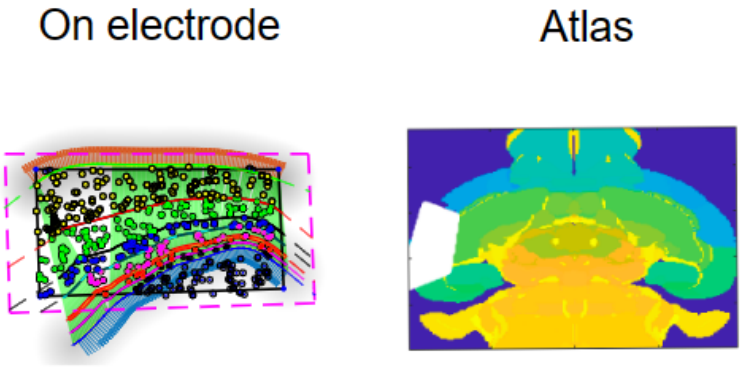

(c)

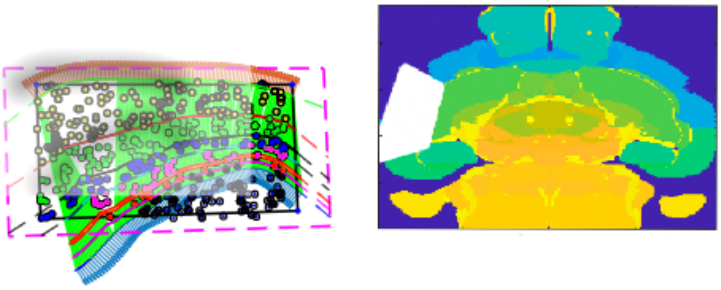

b. Left Occipital Ventral

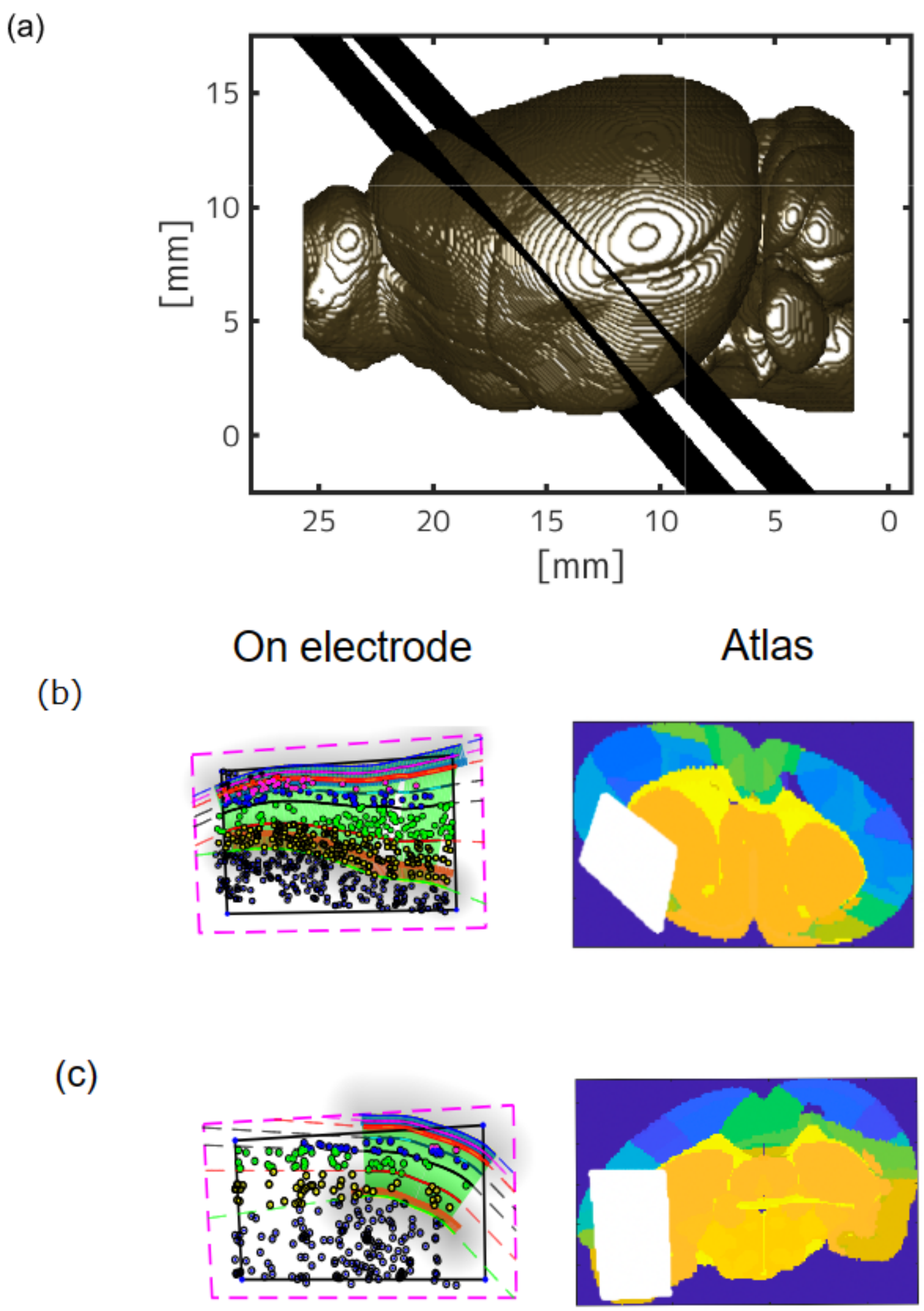

c. Left Ventral

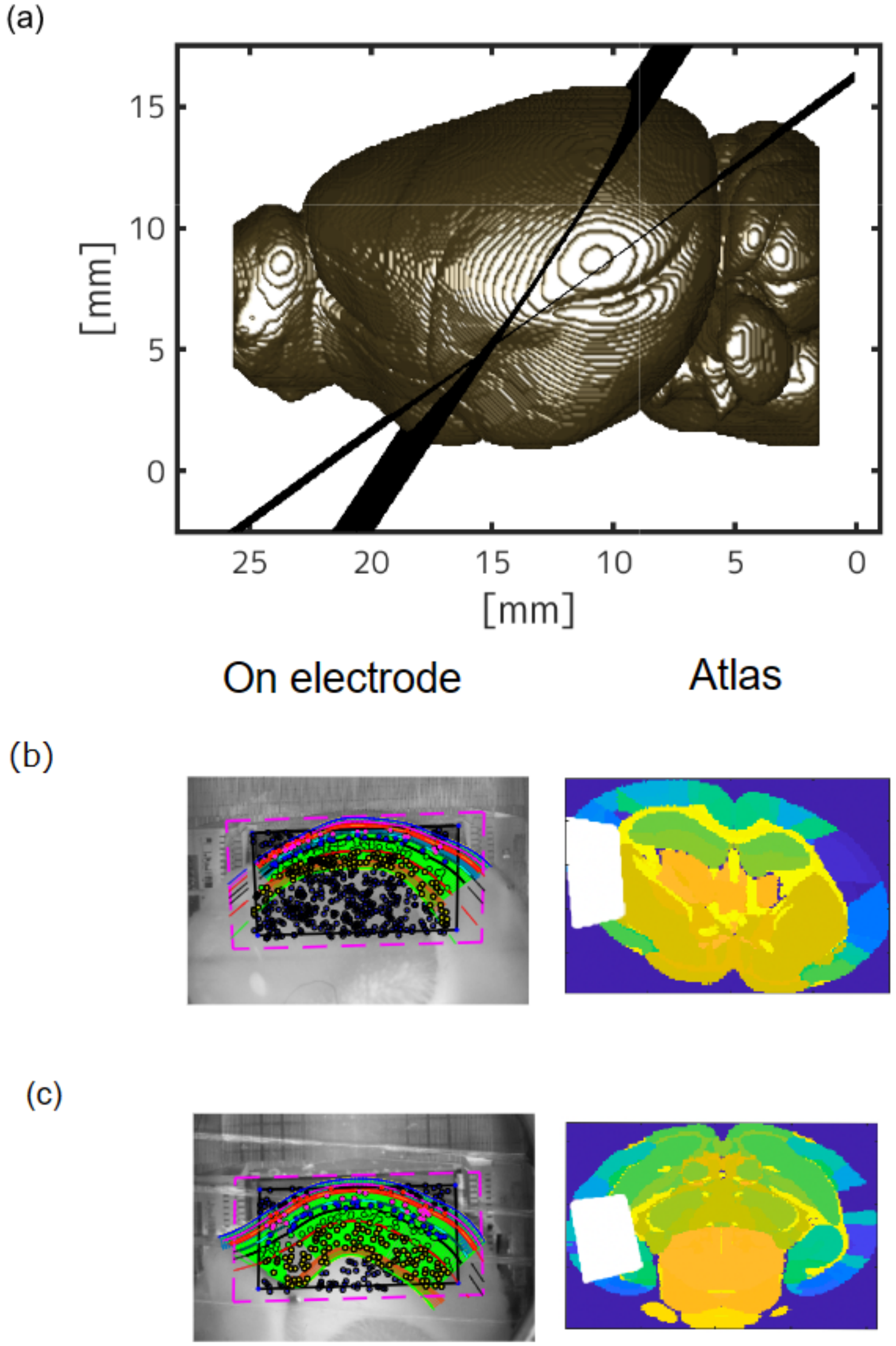

d. Left Frontal Ventral

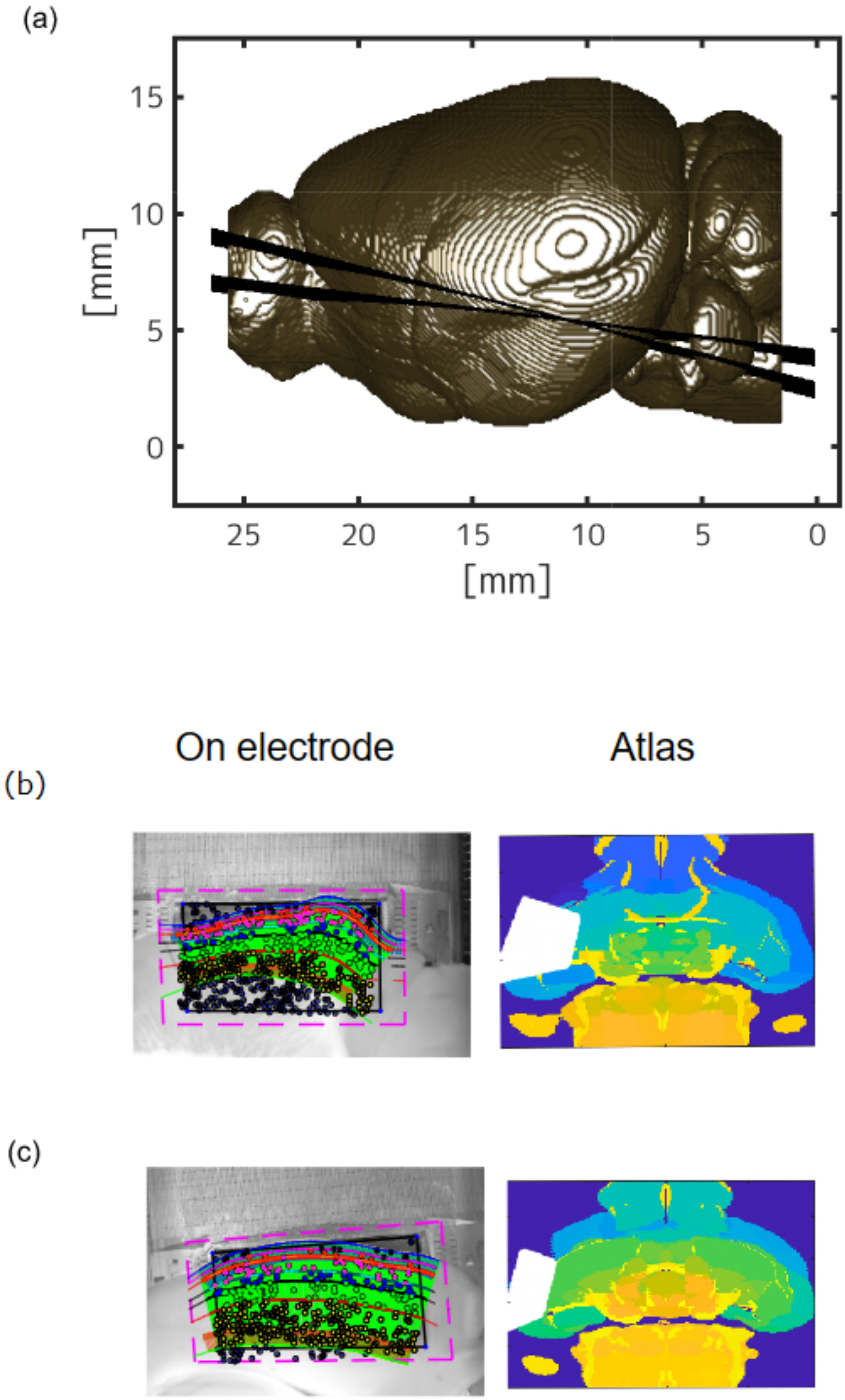

e. Left Frontal

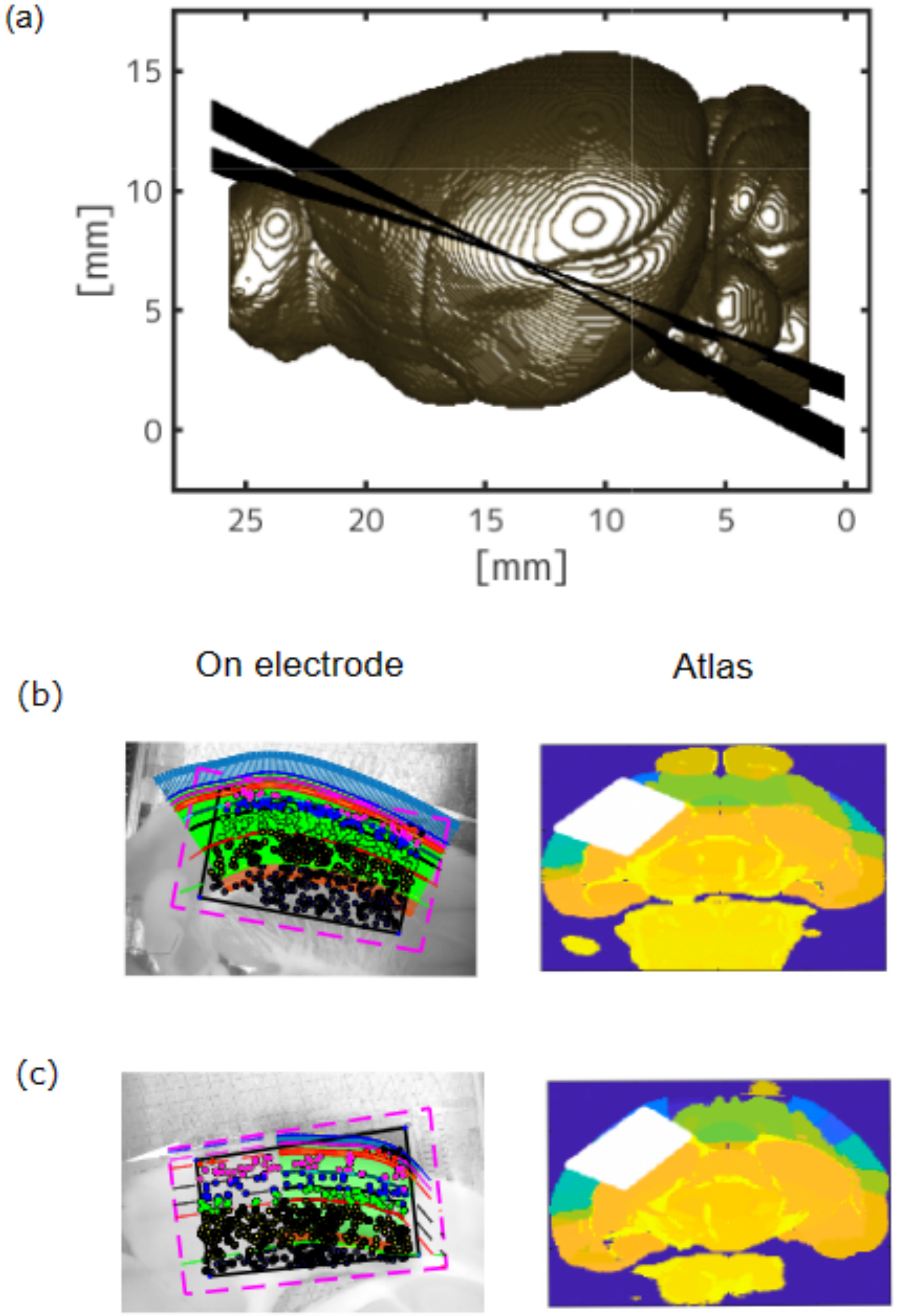

f. Left Frontal Dorsal

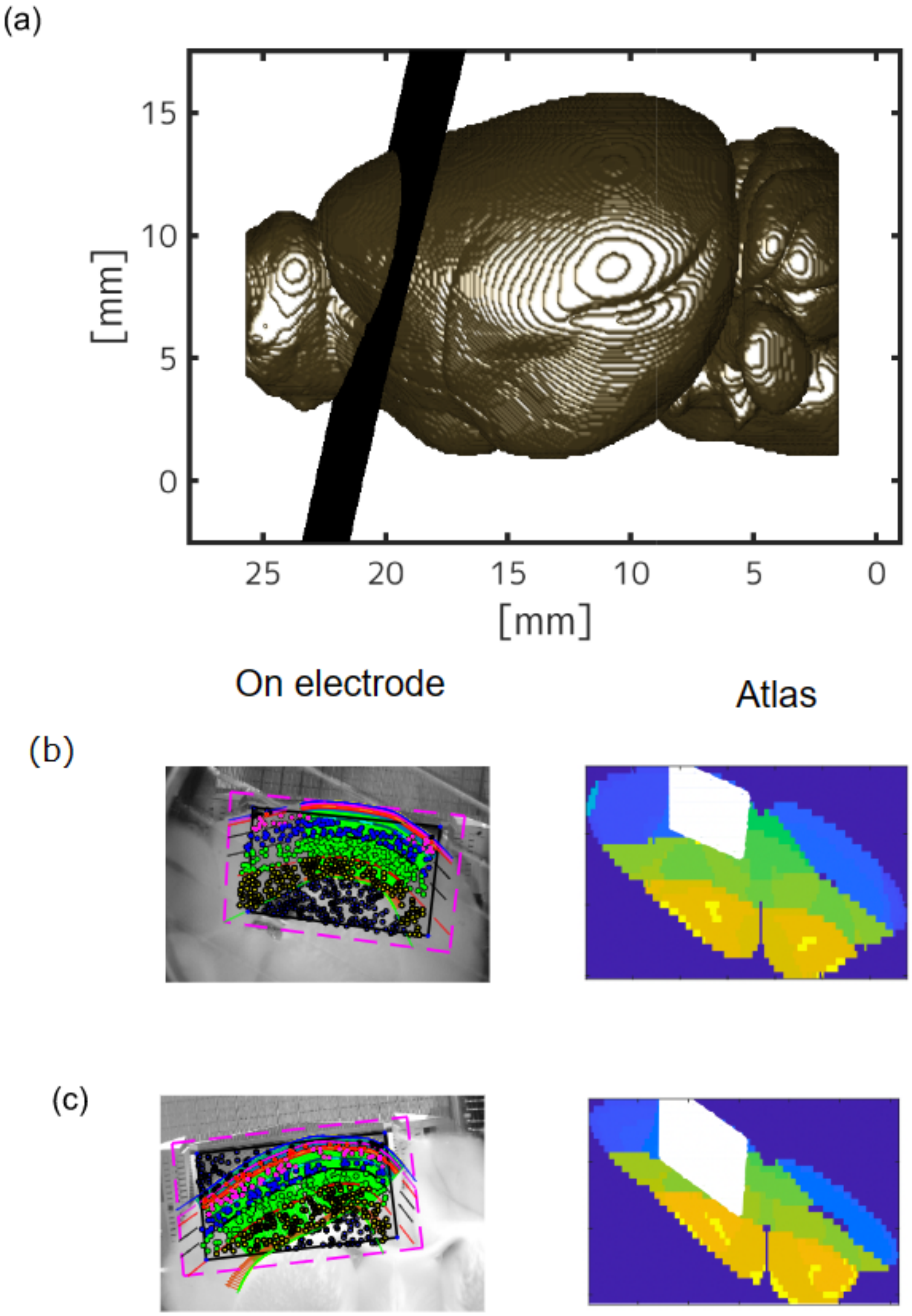

g. Left Dorsal

(a)

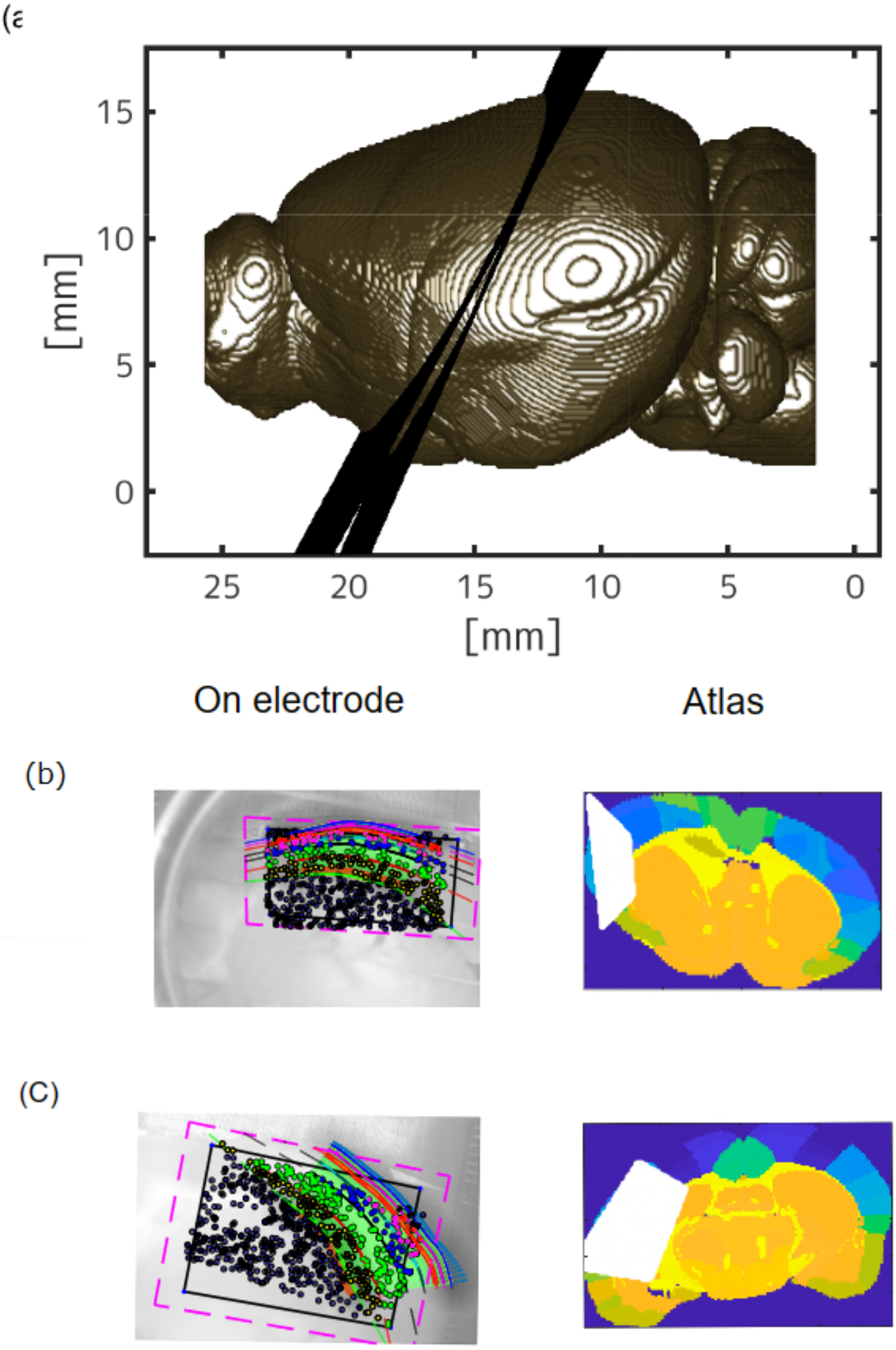

h. Left Occipital Dorsal

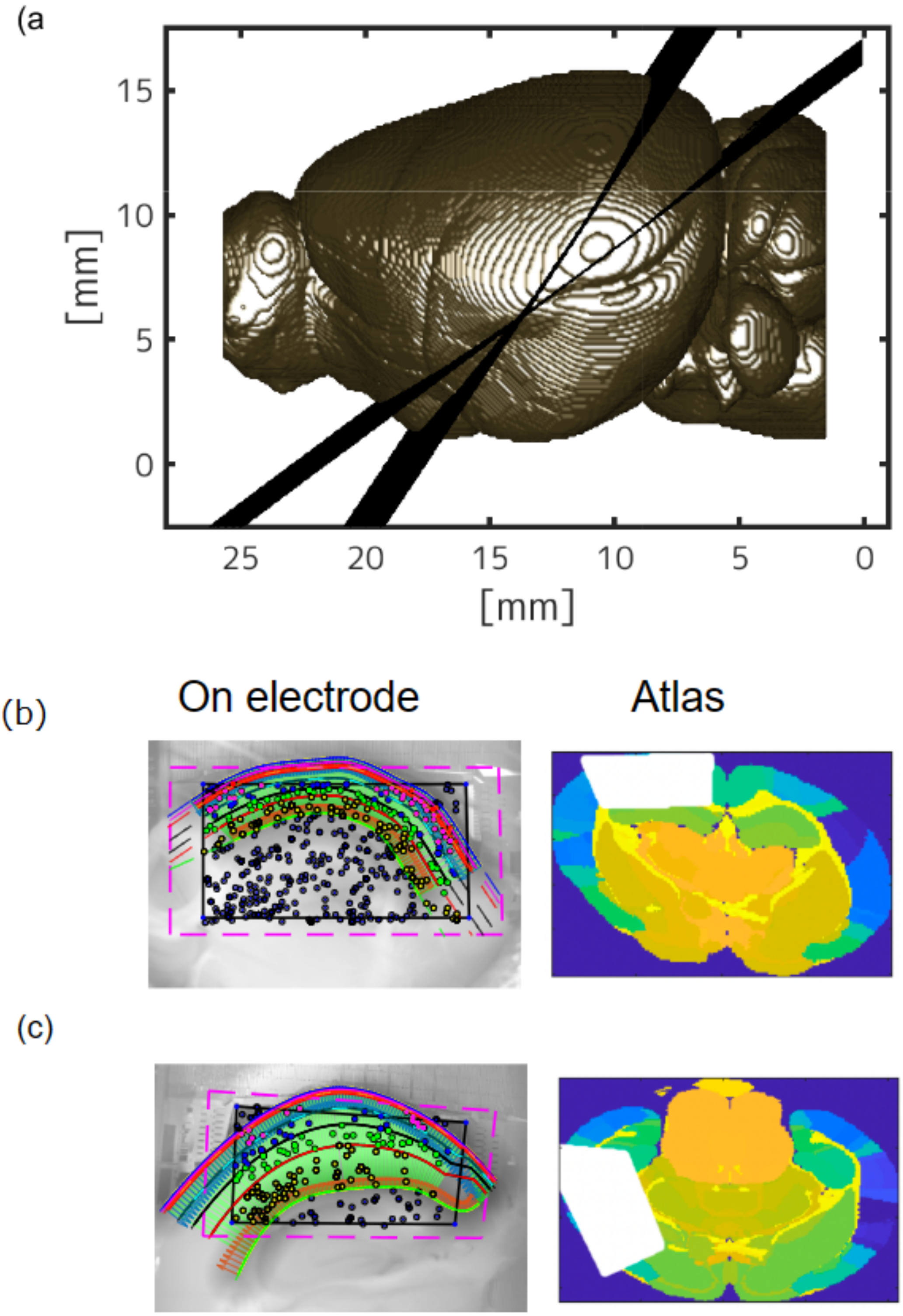

i. Right Occipital Dorsal

(a)

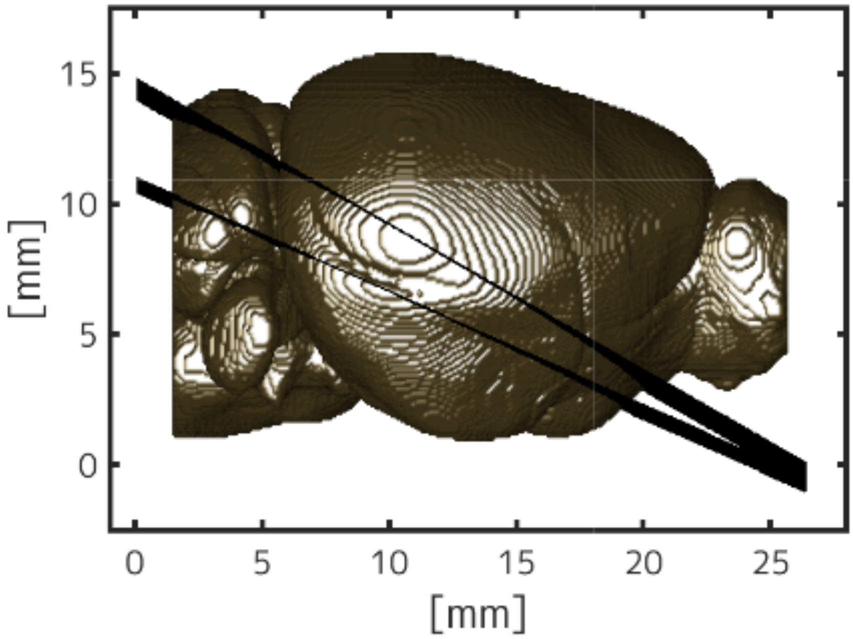

(b)

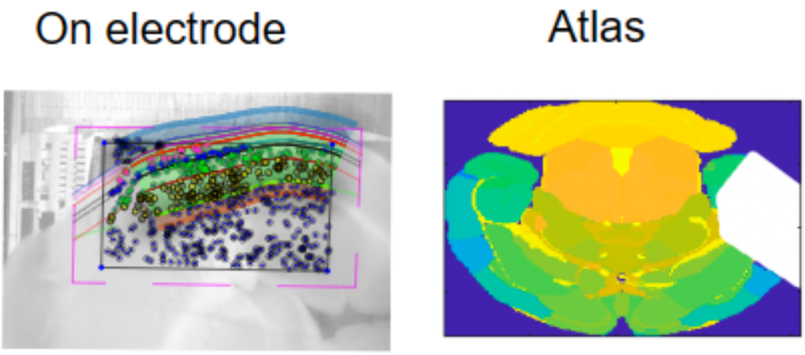

(c)

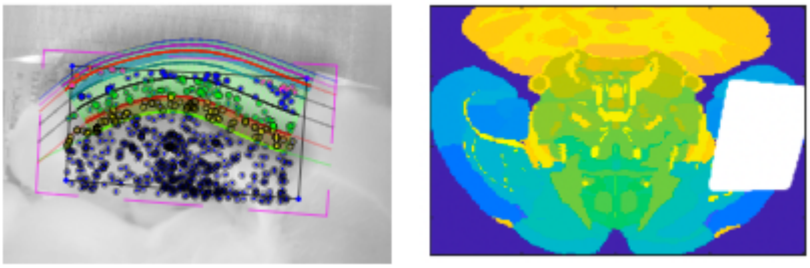

j. Right Dorsal

(a)

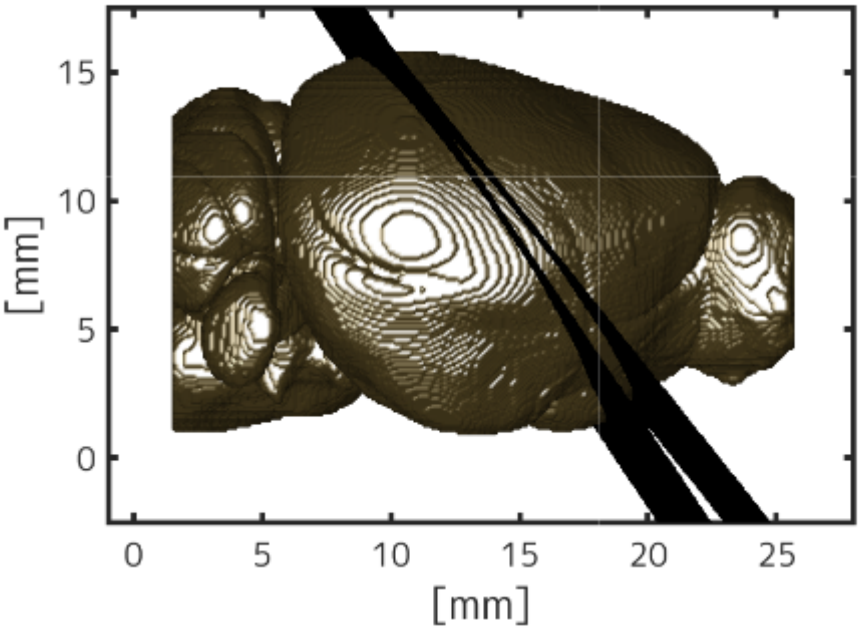

(b)

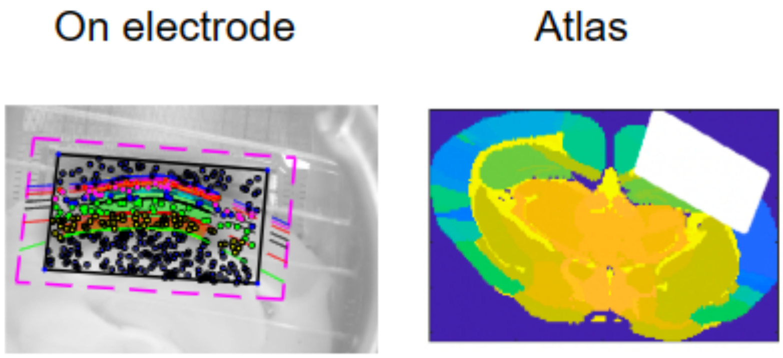

(c)

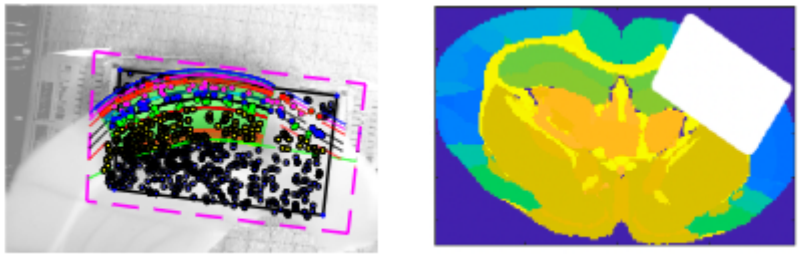

k. Right Frontal Dorsal

(a

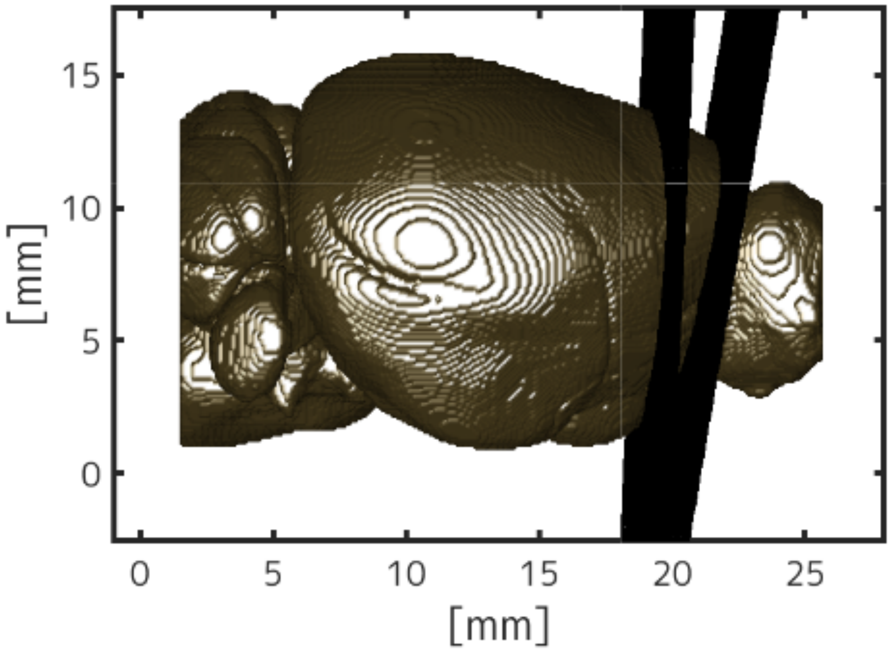

(b)

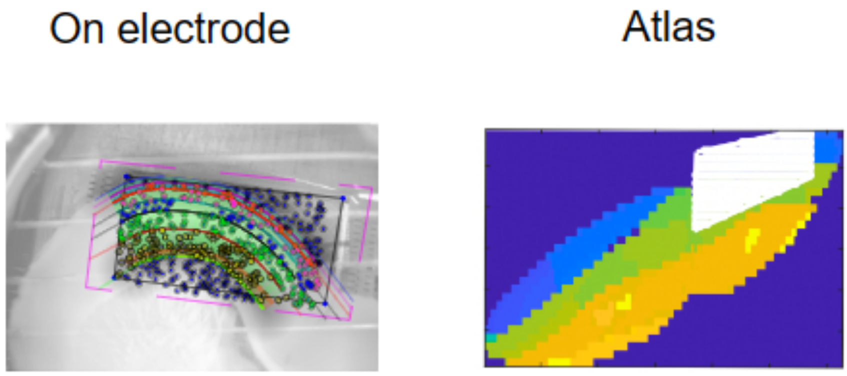

(c)

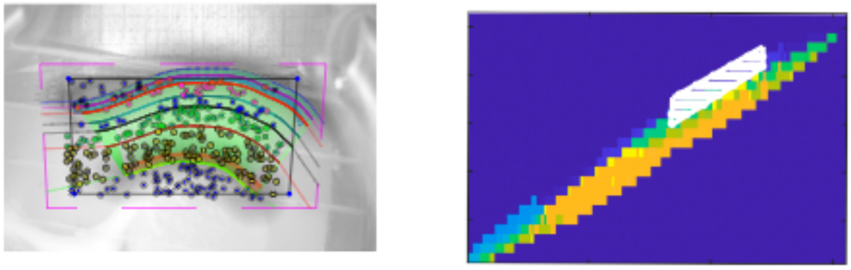

I. Right Frontal

(a)

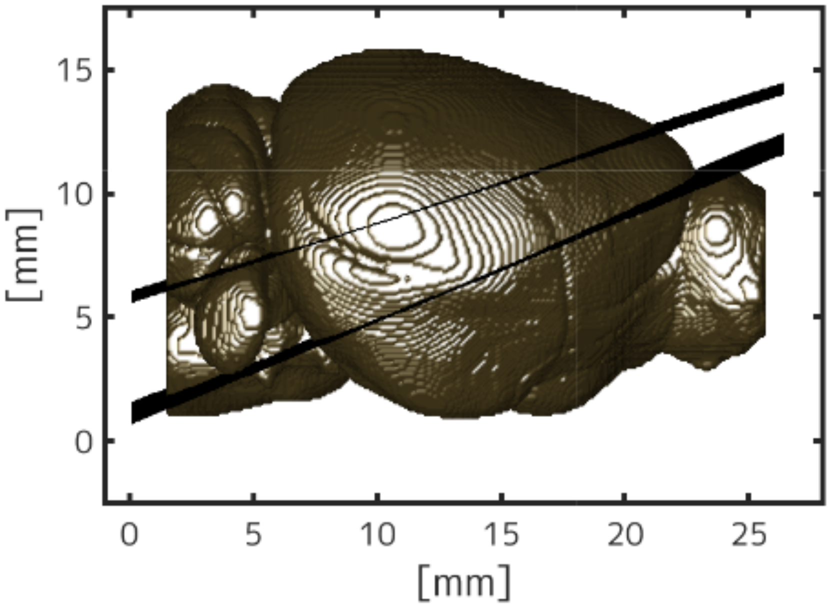

(b)

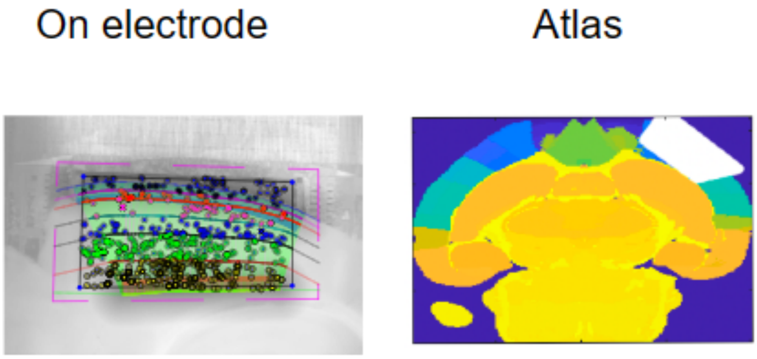

(c)

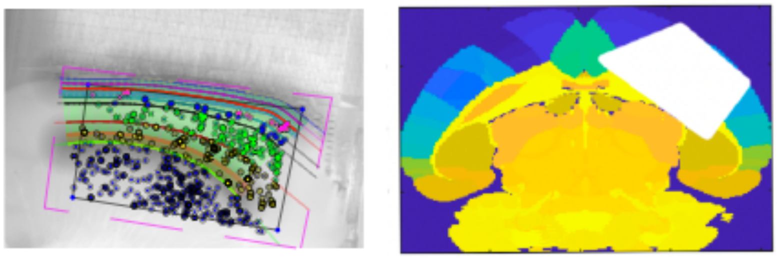

m. Right Frontal Ventral

(a)

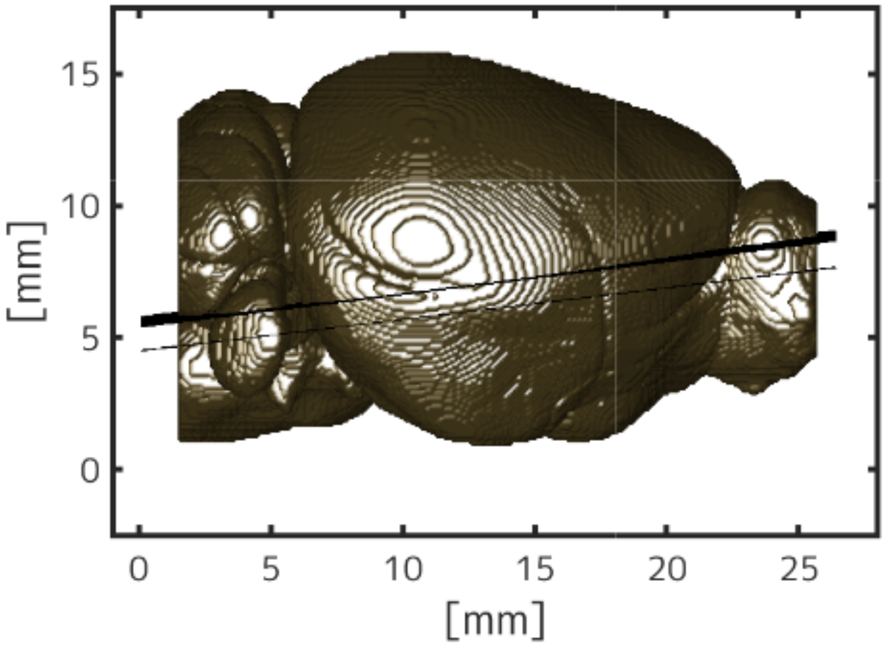

On electrode Atlas

(b)

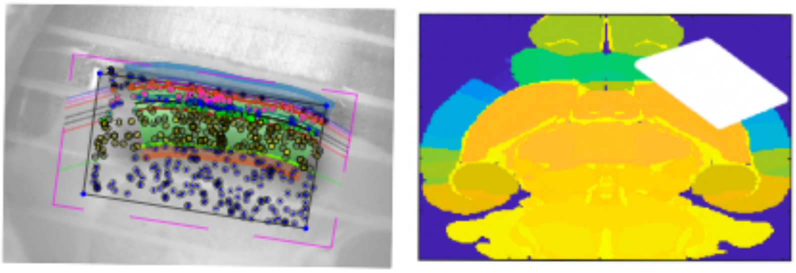

(c)

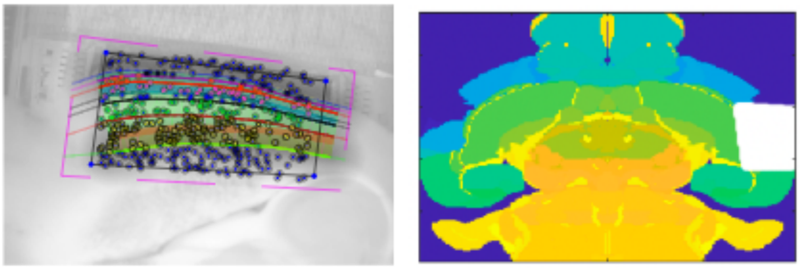

n. Right Ventral

(a)

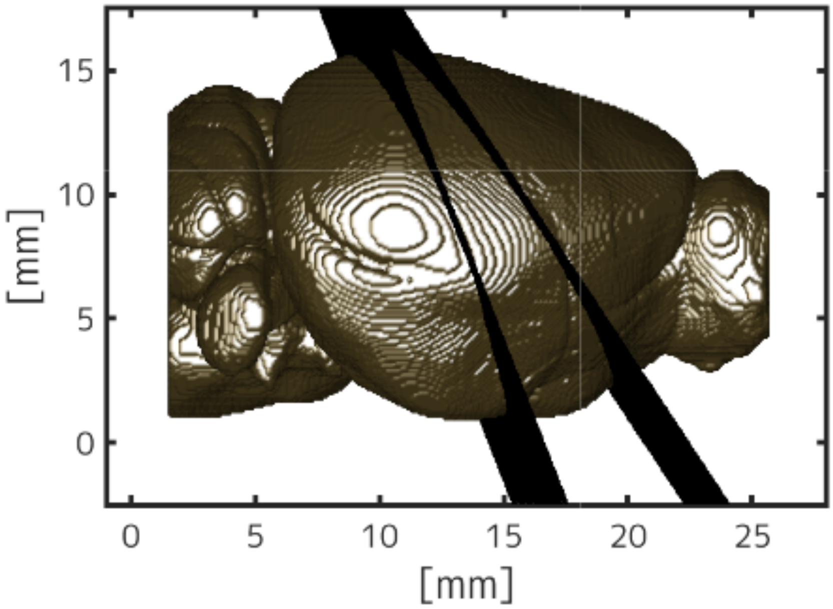

(b)

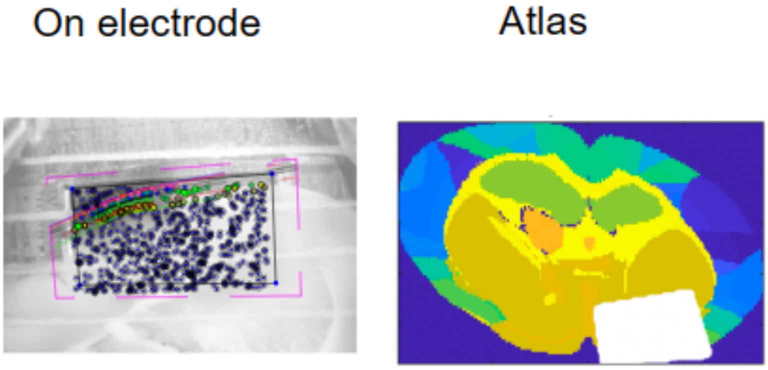

(c)

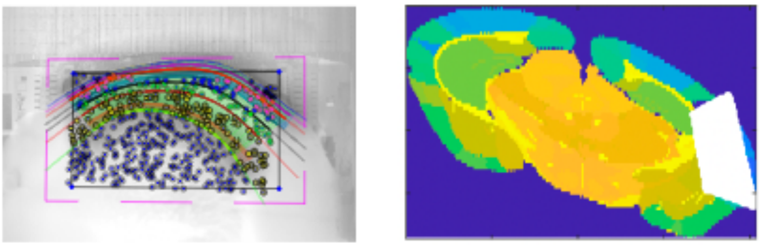

o. Right Occipital Ventral

(a)

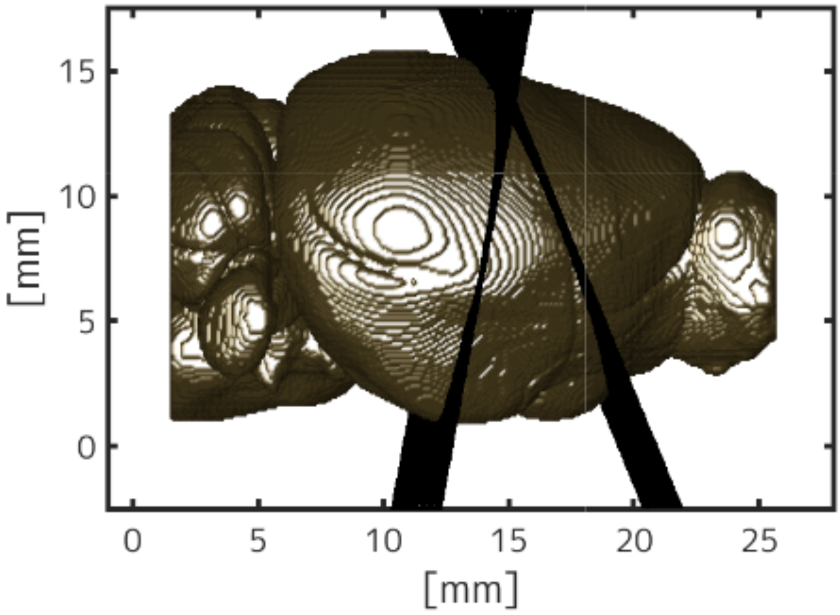

(b)

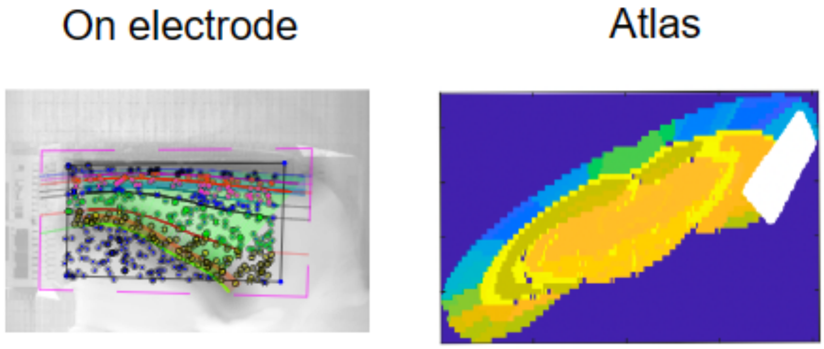

(c)

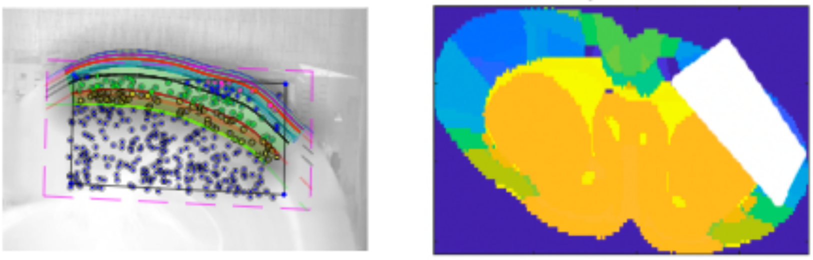

p. Right Occipital

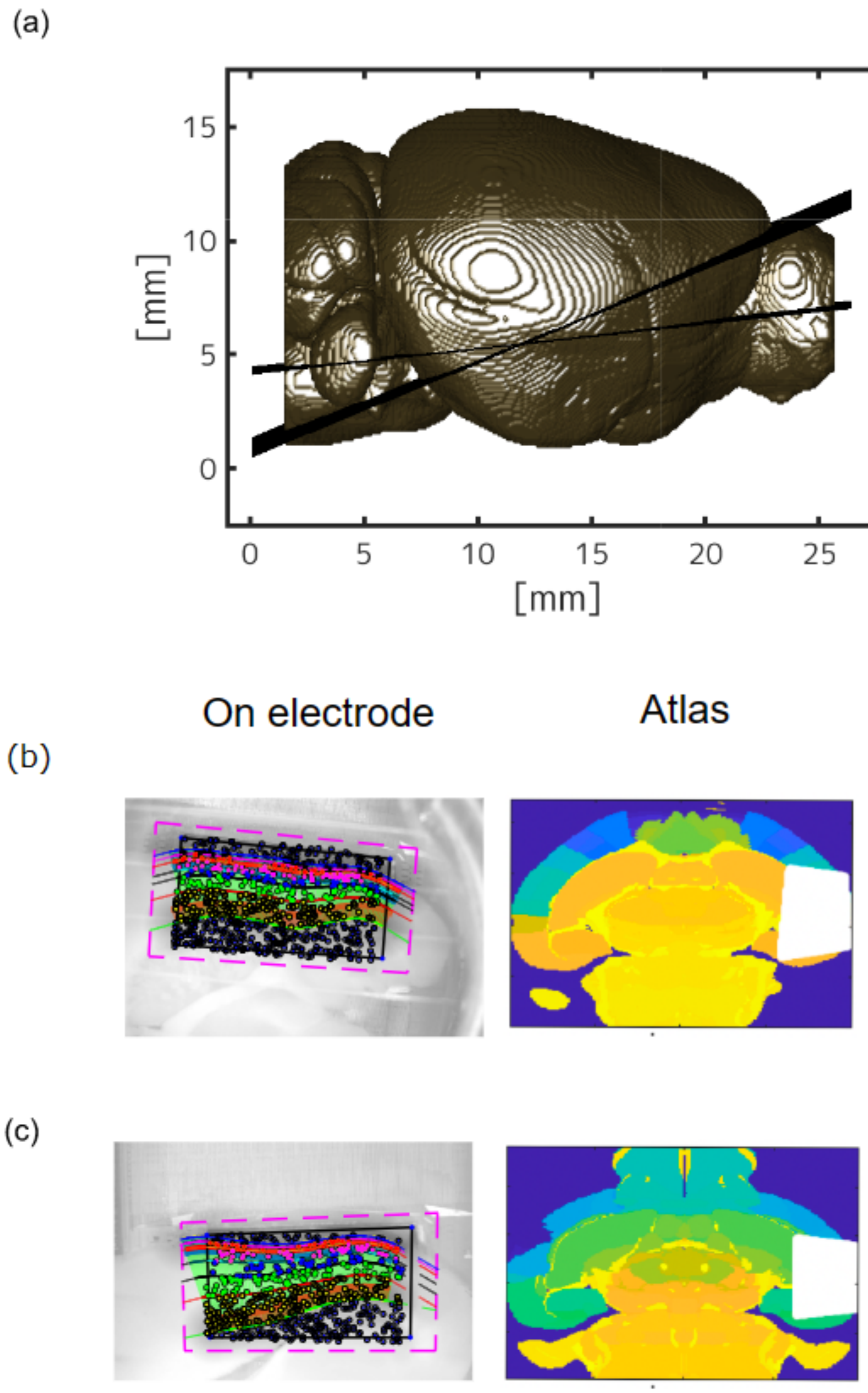

Supplement: Supplementary file 2 — Supplemental Material 1 [file 42003_2023_5453_MOESM2_ESM.pdf]
